# Approved or disregarded? Exploring arenas for narrative relations in geriatric care

**DOI:** 10.1080/17482631.2023.2293130

**Published:** 2023-12-12

**Authors:** Lisa Herulf Scholander, Anne-Marie Boström, Staffan Josephsson, Sofia Vikström

**Affiliations:** a Division of Occupational Therapy, Department of Neurobiology, Care sciences and Society, Karolinska Institutet, Stockholm, Sweden; b R&D unit, Stockholms Sjukhem Foundation, Stockholm, Sweden; c Division of Nursing, Department of Neurobiology, Care sciences and Society, Karolinska Institutet, Stockholm, Sweden; d Theme Inflammation and Aging, Karolinska University Hospital, Stockholm, Sweden

**Keywords:** Geriatric care, health services research, interpersonal relations, interprofessional relations, narration, qualitative inquiry, person-centred care

## Abstract

**Purpose:**

The use of narration in healthcare has been accentuated as a response to the requested shift towards person-centred care. The notion of narrative relations refers to a process of involving several people in mutual and ongoing narrative exchange. This study aimed to explore how and where narrative relations may be adopted and enacted in everyday healthcare practice.

**Methods:**

The study has a qualitative, explorative design. Seven interprofessional focus group discussions with healthcare staff were prompted by vignettes. A multidisciplinary team of healthcare staff (*n* = 31) were recruited on a geriatric ward. Data were analysed using a constant comparative method.

**Results:**

A core theme shows how narrative relations are adopted and enacted both as part of an *approved practice*—the work procedures commonly approved as part of healthcare, and as a *disregarded practice* where covert but important narrative relations take place to support fundamental qualities of healthcare. Moreover, the findings consider arenas of healthcare practice where approved or disregarded practices are enacted in the clinic frontstage and the clinic backstage.

**Conclusions:**

Narrative relations may take place in different arenas of healthcare practice yet simultaneously become a cohesive force interconnecting those arenas and uphold continuity. Impeded narrative relations in one arena may have unintended consequences in another.

## Introduction

As various narrative aspects of healthcare practice have been highlighted in response to the call for integrated and people-centred healthcare (Buckley et al., 2014; Clark, 2015; Ekman et al., 2011; World Health Organization [WHO], 2015) several frameworks building on narration to translate philosophies of person-centred care into practice have been developed and researched (Britten et al., 2017; Buckley et al., 2014; Charon, 2001; Ekman et al., 2011). Although these frameworks relate to various ontologies and epistemologies, and have different implications for practice, they adopt narratives as fundamental in creating meaning and coherence out of human experiences (Polkinghorne, 1988; Ricoeur, 1984). Although there is not one generally accepted definition of narrative, a general feature of narrative is the notion of emplotment, i.e., the organizations of the events or actions into a plot which is used to create meaning of human action and experiences by configuring heterogeneous elements into a temporally meaningful whole (Ricoeur, 1984). Narration refers to the activity of creating narratives, whether in action or words. As such, narratives and narration may offer insights into the meanings people create around their experiences, life situations, events or illnesses. Recent studies on the role of narration in person-centred practices (Fox & Brummans, 2019; Josephsson et al., 2022; Scholander et al., 2021, 2023), have suggested a focus shift from narratives as merely verbal and individual accounts, towards the narrative form of everyday action and meaning-making and how narratives are embedded and co-constructed in social interactions. Still, despite these theoretical advances, healthcare professionals (from hereon called staff), key players in translating frameworks and theory into everyday practice, may not link their everyday actions to theoretical concepts such as narration or person-centredness. The same applies when aiming to translate everyday meaning-making and action into theory and professional procedures. There is a tension between how everyday experiences and theory communicate.

Geriatric care is one specific area of healthcare where person-centredness and narrative practices have been suggested to be particularly important (Berendonk et al., 2017; World Health Organization, 2015). A preceding qualitative study on a geriatric care ward (Scholander et al., 2023) explored how the use of narration was reflected in staffs’ experiences of everyday practice as constructed in interprofessional focus group discussions and contributed new insights on narrative in healthcare practices by shifting focus from patient narratives as individual products to the ongoing relational processes of narration, in the study called engaging in *narrative relations (NR)*. In contrast to frameworks emphasizing elicitation of patient narratives (Doran et al., 2019; Ekman et al., 2011), the findings showed how NR was reflected as a transactional process involving several people, both patients and staff, in mutual and ongoing narration that functioned to uphold several qualities of healthcare practice, such as preventing simplistic understandings, supporting continuity, peer learning, and relationship building, both between patient and staff and among the staff in this healthcare context. Some of the foundational qualities supported by NR referred to patient-professional mutual interactions, while others related to the interprofessional team interactions. Thus, the NR seemed to take place in different relational contexts of practice and may matter differently in those contexts, which was not further analysed within the scope of that study. Using the term *relational context* here denotes an interest beyond merely physical places where NR take place, but also in terms of groupings, fora or other social interactions between people.

Other studies (Ellingson, 2003; Fox & Brummans, 2019; Lim et al., 2020; Waring & Bishop, 2010) have used a conceptual distinction between the *clinic frontstage* and *clinic backstage* of healthcare practice as one way to demarcate such relational contexts, building on terms originally coined by Goffman (Goffman, 1990). Simply put, in these studies the clinic backstage refers to the interactions and activities off-limit to the patients and including staff only, yet, not necessarily in physically private areas as referred to in Goffman’s original backstage concept. The clinic frontstage refers to the activities and interactions between patients, relatives, and staff. Applying such divisions as a theoretical lens may be useful to achieve a more nuanced understanding of NR in different relational contexts of geriatric healthcare practice, and to get insights into how these parts of practice relate to each other. Previous findings indicate that NR have different implications in various clinical situations, thus how and where they are enacted in everyday practice, and how that matters for person-centredness is yet to identify. Furthermore, the tension between NR as part of professional procedures and NR as part of personal everyday meaning-making merits further exploration. Therefore, the aim of this study is to explore and develop knowledge on how and where NR are adopted and enacted in everyday practice on a geriatric ward.

## Methods

### Research design

This focus group study has a qualitative, explorative design grounded in a constructivist epistemology. We have applied methodological principles from Charmaz’s more recent versions of Constructivist Grounded Theory (CGT) (Charmaz, 2014), since the principles of iterative and parallell data generation and analysis, theoretical sampling, and theorizing grounded in empirical data were deemed appropriate for our purposes. However, beginning accordingly with an inductive analytical approach to create codes and tentative themes, we applied abduction as the main guiding principle, using empirical and theoretical resources to reason and develop the emerging findings in relation to the aim of the study. Here, we follow arguments presented by Timmermans and Tavory (2012), along with Clarke et al. (2018), who advocate the abductive logic as part of theorizing in qualitative research.

Ethical approval was obtained from the Swedish Ethical Review Authority (reference number 2019–00248.

### Data generation and analysis

The study was conducted on an inpatient geriatric ward in a large Swedish city and was part of a larger explorative research project aiming to develop a deeper understanding of narration as a potential resource for person-centredness in geriatric care. One unit was selected to gain a deep and contextual understanding of everyday interactions situated in the local care culture. The ward had a capacity of 42 hospital beds and offered medical care and rehabilitation to older people with recently deteriorating health situations or rehabilitation needs requiring a multidisciplinary team of staff.

The data generation and analysis, conducted as a parallel and iterative process (Charmaz, 2014), build on seven interprofessional focus group discussions with staff working on the ward. Our intention with this method was to create a forum for participants to jointly inquire into the conditions of their everyday practice (Kamberelis et al., 2018) in relation to the use of narration and person-centredness. Moreover, we considered focus groups suitable to gain insight into the collective and communicative processes of reasoning assumed to shape staff’s understanding of everyday practices on the ward.

All staff working on the ward, at that point nearly 100 employees, were eligible for participation, informed about the study and invited to participate via email, written information on noticeboards, and oral presentations during regular staff meetings. One third of the staff, i.e., a total of 31 participants chose to participate and gave their written informed consent. The sample was diverse and multidisciplinary, including both of staff and unit managers (see Table I), and the number of participants from each profession reasonably reflected the staffing ratio on the ward. The focus group composition was strategically planned to achieve interprofessional representation from at least two different professional groups and ranged from two to four represented professions. A large proportion of participants, 87%, were female and 13% were male, reasonably reflecting the gender distribution on the ward. The discussions, led by the first and last author, took place in a conference room at the hospital. Discussions lasted 77 min on average and were audio-recorded and transcribed verbatim. The first author was familiar with the care environment from conducting participant observations on the ward in 2019 (Scholander et al., 2023). Hence, several participants were familiar with the research project and the researchers’ interest in narration in relation to person-centred practices.

**Table I.** Participants and basic demographics.

Profession	n=32^a^	Gender, Female/Male/ Non-binary	Work experience in current profession, Range in years (median)
Nursing assistant	11	11/0/0	0.6–34 (5)
Nurse	9	8/1/0	0.3–26 (3.5)^b^
Physician	4	2/2/0	1–4 (1.3)
Occupational therapist	3	3/0/0	1–27 (10.5)
Physiotherapist	2	1/1/0	5–24 (14.5)
Unit manager	3	3/0/0	5–6 (5.5)^b^

^a^31 unique individuals. One participant attended twice: first as a nurse, later in a new position as unit manager.

^b^Imputed one missing datum.

A vignette portraying an everyday situation of patient and staff interaction on the ward was used to prompt discussion. One vignette was developed from a previous ethnographic study on the ward (Scholander et al., 2023). In the final group, we used a vignette built around a salient theme emerging from the analysis related to staff’s interactions and interpretations of each other’s actions. The first and last authors moderated the focus groups and used questions and follow-up questions to cover broad topics of interest for the study, outlined in an interview guide, yet constantly developing in line with the methodological principles of CGT (Charmaz, 2014).

A constant comparative method, moving back and forth between data generation, data coding, and emerging themes guided the analysis (Charmaz, 2014). Emerging questions were further explored in the latter focus groups in line with the CGT principle of theoretical sampling. Throughout the research process, participants were asked to reflect on emerging analytical ideas. Memo writing was employed throughout the whole process as part of the analytic work. Eventually, theoretical resources were brought in and used abductively to enrich the insights emerging from the empirical material, while the empirical material simultaneously could add new nuances to the existing theoretical concepts in this context. This resulted in new conceptual ideas advancing the interpretation and understanding of the different relational contexts where NR took place. We regularly held analytic discussions between the four authors who contributed with their perspectives by representing three healthcare professions and various research backgrounds.

The data, at just above 87,000 words, contain vivid, contextual descriptions of participants’ experiences and reflections on everyday situations in healthcare practice and were considered rich enough for further in-depth analysis in relation to the research focus. Based on the study’s epistemological position we view the data and findings as situated and constructed which requires rich description about context, multiple examples in the data supporting the presented findings, and an absence of data that contradict the presented findings. At the end of the seventh focus group, all authors agreed on that these criteria were adequately met. The constant comparative method together with the iterative process and theoretical sampling helped us to safeguard rigour in this sense. To manage the data, we used the Atlas.ti software.

**Figure 1.**
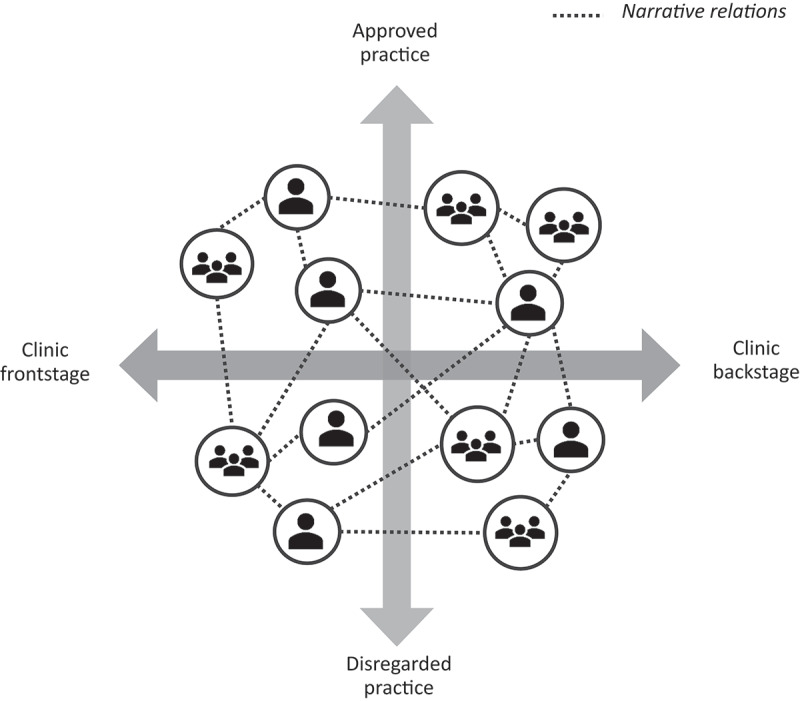
A depiction of four arenas of practice and NR defying or being challenged by the boundaries between these arenas.

The findings are reported in two parts. Firstly, a core theme that repeatedly arose from inductively approaching the data is presented: a twofold practice where NR were adopted and enacted was identified, yet the two parts were construed as positioned on a continuum. Secondly, we developed a graphical illustration (Figure 1) by intersecting this continuum with an axis representing the clinic frontstage and clinic backstage, here also construed as a continuum corresponding to the core theme. By so doing, four main arenas of practice appear as a visual representation serving to frame the findings.

#### Research team and personal characteristics

Four researchers (gender distribution F = 3, *M* = 1) represented various academic backgrounds and professional disciplines: the first author physiotherapist, MSc, PhD-student; the second author registered nurse, associate professor; the third author occupational therapist, professor; the last author occupational therapist, PhD. All authors were experienced in various qualitative research methods. Author contributions are specified at the end of the paper.

#### Analytical resources

Our philosophical foundation in narrative theory offered an expanded understanding of narrative not restricted to verbal stories, but acknowledged the connection between narrative, meaning-making and action (Josephsson et al., 2022; Mattingly, 1998; Ricoeur, 1984). The analysis was guided by the notion of *narrative relations* (NR) developed in a previous study (Scholander et al., 2023). NR refer to the idea that narration in healthcare practice is characterized by a mutual and ongoing narrative interchange between multiple narrators, including patients, their relatives, staff and external actors. Recognizing this as a sensitizing concept rather than a definite concept (Blumer, 1954; Bowen, 2005) offered a way of seeing that guided further analysis, rather than a settled definition. As such, it may support the process of theorizing towards achieving a situated understanding of how NR are adopted and enacted. Additionally, from our current position narration is understood as a communication capability and activity that staff and patients use to pursue and uphold foundational qualities of healthcare practice.

We applied the conceptual division clinic backstage and clinic frontstage as used in other healthcare studies (Ellingson, 2003; Lim et al., 2020; Waring & Bishop, 2010), thus not only referring to physical locations but also including interactions and interpersonal relations. We used this division as a heuristic device for moving into further analysis (Clarke et al., 2018; Timmermans & Tavory, 2012). Although useful to start by chiselling out different relational contexts of practice, we consciously strived to maintain a critical distance to these concepts, avoiding using them as a merely deductive means to organize the data. Instead, we strived to continuously stay open to grey areas or new possible differentiations.

## Findings

The core theme, *Hidden or approved—narrative relations traversing a twofold practice*, demonstrates a twofold practice in which NR are adopted and enacted, yet simultaneously become a cohesive force interconnecting those practices. The core theme is substantiated by three subthemes and illustrated by quotations from the data, identified by pseudonyms for each participant. Tensions identified in each subtheme are further described in the main text.

When the continuum between approved practice and disregarded practice was intersected with the continuum between clinic backstage and clinic frontstage, derived from theoretical resources, four arenas of practice emerge which create a frame for our findings, helping to elucidate implications for practice (Figure 1). This means that the approved and disregarded practices may occur both in the clinic frontstage and in the clinic backstage.

## Core theme

### Approved or disregarded—narrative relations traversing a twofold practice

The analysis showed that some clinical activities were recognized by staff as generally accepted work tasks forming the part of practice we have chosen to call the *approved practice*. Other activities were often disregarded and carried out in hidden ways. We have chosen the term *disregarded practice* for this part of practice. The activities of the disregarded practice were rarely seen as formal procedures but nevertheless perceived by staff as adding significant value and quality to the work such as achieving continuity in the practices or fostering peer support and learning from colleagues. This distinction is substantial for the findings since NR could be more or less acknowledged in the approved practice. Engaging in NR was identified as an often oblivious and tacit activity of everyday work, hence part of the disregarded practice. Yet, engaging in NR could also be part of formalized fora or routines building on narrative activities and exchange, thus becoming part of the approved practice. When obstructed, the NR may be transferred, obliviously or deliberately, to a disregarded practice to meet needs that are not covered by the approved practice. However, while imbuing both parts of practice, NR make them communicate. The tensions and movements between approved practice and disregarded practice is demonstrated in the subsequent report.

The core theme is substantiated by three distinct yet interconnected subthemes emerging from the analysis. Since we consider their interconnection more relevant for the study purposes, we have made the choice to report them as embedded in one coherent text, instead of under separate subheadings. As a supplement, subthemes and their inherent tensions are specified in Table II.

**Table II.** Core theme, subthemes, and tensions within the subthemes.

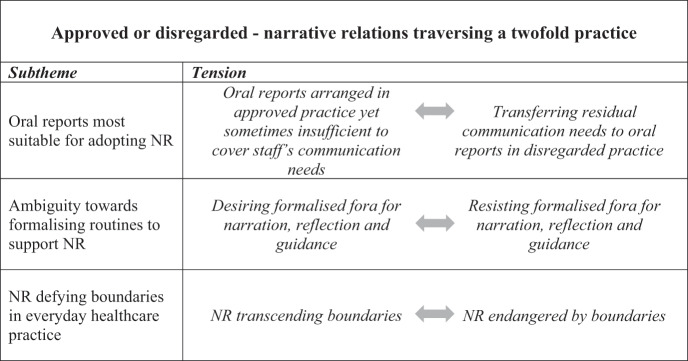

In the approved practice, written accounts—particularly patient records—were frequently referred to as a primary and reliable tool for transferring information about patient situations, performed procedures and healthcare objectives. However, to adopt NR, oral reports and narrating particular matters were used as a complement to reading the records. Oral reports were arranged a few times a day in the various staff groups, while interprofessional team rounds were scheduled once every morning. Through oral reports, underlying reasoning and motives to actions could be communicated in a way the records could not, as exemplified by the physician, Michael: *‟ … but to say something orally is also like … It will be easier for the other person to understand than if it just shows up as a urine sample [in the records]/ … /And if I just write it like a to-do-thing urine sample, then it may become difficult for the person who does not know the reason.”* Also, certain information was narrated orally as it was considered unsuited for the records. The nurse Amanda said: *“At the morning meeting/ … /you bring up things/ … /which are not suitable to write in a patient record. Some information is good to communicate but is not suitable for the records, such as how someone prefers to have their juice placed on the table.”* Similarly, written referrals covered basic information, but particular matters about a patient were better told orally as exemplified in the dialogue between OT Alicia and PT Johanna, indicating needs of communicating in more informal ways: *“Alicia: It’s a bit different too, I think, sometimes in the referrals […]you just write what is needed in the referral to the home rehabilitation unit, but if there is something particular about the patient/ … /Johanna: Then you call. Alicia: … then you call so/ … /they get a different picture; it may have been a difficult hospital stay … so it could be good to report that so they do not get a surprise when they get home.”* This type of information often relates to personal or ambiguous circumstances, either regarding staffs’ experiences or more sensitive patient matters, and is thus important for person-centred practice.

Our analysis showed how oral reports and handovers were primarily conducted within separate professional groups, and the narrative content appeared as prevented from crossing professional borders, except during team rounds. However, there were not always formal procedures for facilitating this focus, but often a checklist-based, medically-oriented format. The shortness of such arrangements often seemed to contribute to disrupted NR between staff groups. However, separate professional groups tended to continue these conversations after the team rounds to cover everything missed during the formal meeting, hence, transferring it to a disregarded practice. So, although staff groups were engaging in NR, there was a tendency towards NR not being sufficiently supported to cross professional borders. Hence, NR seemed challenged by the various boundaries in everyday practices. A similar example was the constructed boundaries between medical and social information. Recorded patient narratives could easily be overlooked or deliberately disregarded in favour of medical information, as illustrated in dialogue between a physician, Victor, and a nurse, Kim. *“Victor: It’s just a strength of course [to read nursing assistants’ and nurses’ notes]. But we do not always use it./ … /Because, yes … there is/ … /a lot of social problems for patients and, like especially the doctors, just want to focus on the medical. So often we just read our own notes to … well./ … /Kim: I will never forget … there was a surgeon who once said to me, “to read nurse notes it’s like opening Pandora’s box, it … [Laughter] You don’t know what you’ll find” … / … /It’s a bit like that at times.”* The quote indicates a dualistic conception of health, distinguishing between the biological and social. Here, only the medical aspects were part of the approved practice according to the physician, while the social aspects were part of the approved practice from the nurse’s perspective, showing that approved practice is not static but may vary between professions. Also, it demonstrates how the fluid boundaries between clinic frontstage and backstage occur, and that narratives may cross boundaries between different parts of practice yet become disrupted en route.

The activity of achieving continuity and transferring both information and narratives orally among staff is described by OT Alicia as *“a continuous ongoing conversation”* involving different professions, both in and outside the ward. Although descriptive information and emplotted narratives are qualitatively different ways of communicating, they are also interrelated in that the NR pave the way for cooperation and for transferring and interpreting information. Participants described how this continuous conversation needed to be embedded in the work culture, which in turn influences the quality of the teamwork. When the NR were disrupted, cooperation in the team was affected. A quote from nurse Hannah indicates the importance of ongoing communication in the team: *‟I’m thinking of teamwork here and what culture you have at work. It is important to know how we go about it. Who should inform, do we speak the same language, for example/ … /so it will be sort of good, like continuity. So that everyone says the same thing and you know who to refer to if the patient has questions. And like, “I don’t know much about that [topic]but about this and that I do … ” So, here, good teamwork may help … ”*. This is also an example of the transboundary function of the NR, bridging the clinic frontstage and backstage. Seemingly trivial things regarding someone’s everyday routines, like how they prefer to take their medications, or what they like to wear when going to bed, could easily be overlooked with a task-oriented focus. The often-disregarded practices of communicating details and preferences may contribute to give patients a sense of trust that staff are continuously informed about their situation and that the care will not fall short.

The analysis shows several efforts to create structured arrangements where narration was a central characteristic and part of the approved practice, such as collegial reflection groups with various purposes. However, those efforts often faded over time, while informal narration showed to be more persistent. On the other hand, enacting the NR as a disregarded practice made it dependent on individual initiatives, hence dependent on personal factors, organizational hinderances and time constraints. Appreciated efforts had been made to create fora for ethical reflection, discussing particular cases or team cooperation, including recent contributions handling uncertainties during the COVID-19 pandemic, as exemplified by two unit managers: *“Erika: During the pandemic, we had a lot of reflection meetings because it was … It was completely new, not only to us but to the whole world. What is this … ? Why … ? So, it was one message in the morning and another message in the afternoon. Then you are forced to do something good for your staff so every afternoon we sat in a large group, doctors, allied health staff, all the nursing staff and just, okay, how have you been today? What did we learn today when we did something right? And when did we do something wrong. We do not do that again tomorrow, but we just take the right thing with us and then it became an affirmation that nursing staff … or healthcare staff … they can really carry out quality work because they do it from one day to another, or from morning to afternoon. Wow wow wow! Pauline: This could really be seen during the pandemic. Erika: Great praise [to the staff], and do not say that quality work takes time, because I do not agree with that.”*

Our analysis showed how participants tended to plead for one of two poles, either advocating formalization and creating routines, or promoting the necessity of keeping the disregarded practice informal, yet seldom discussing a possible middle ground where the disregarded practice itself may be approved as a vital part of practice that requires adequate room in everyday work. However, they enacted a middle ground in some routines and formalized activities which were initiated bottom-up by some professional groups. The OT Alicia described the allied health staffs’ morning meeting, where the conversation climate also allowed for debriefing and peer support: *“We generally meet daily in our paramedic group to talk about things like … everything that happens in the ward and if there are patients who are more demanding in some way, so we discuss such things a lot I think.”* A few participants identified the possibility to include NR as part of the approved practice by integrating interprofessional narration about everyday experiences during already-established meetings. As the team rounds were generally accepted by all professions, these were suggested as a feasible and sustainable point of departure. Routine meetings already allowed for this, to some extent, as shown in the dialogue regarding handover between two nursing assistants: *‟Gabi: So then I say, but I will have the same group as you had, then you tell about each of those patients, what you did and did not do. How she has felt today, if she’s worried, if she’s been very tired—well such things so that the next one who comes know that. Elisabeth: That handover between day and evening usually works well, I think … ”*. The examples above show how staff together create narratives from particular insights regarding the patients’ situations, but also how they integrate their own actions and intentions.

In addition to the few existing arrangements that helped encourage NR as part of the approved practice, we identified an ambiguity towards adding formalized routines building on narration about particular clinical situations. Desired activities included supervision or facilitated collegial group reflection on specific cases or ethical dilemmas. Although identifying a need among staff for regularly venting their feelings and frustrations emerging from everyday work situations, the overall argument against the idea of creating structured arrangements was that it would be too distant from the original event to be relevant for handling the emerging feelings and questions that the fora aimed to help deal with. Rather, the needs for support and guidance typically arose from particular situations and demanded to be met close to the situation with those involved, as exemplified by the physician Victor: *“Spontaneously I think it is … it is the informal that works. I do not know how a … like structured … It feels a bit forced. You often have those feelings there and then and want to vent them then. It is not often that you take them home and carry them with you for several days to wait for … [a scheduled meeting].”* However, to systematically allow for this informal practice, it must be widely accepted, encouraged and applied—pointing at the grey area between approved and disregarded practice, where giving room for spontaneous and informal interactions may ultimately become part of the approved practice. Also, analysis showed that despite the lack of structured fora in approved practice, participants frequently praised the open climate on the ward that allowed for seizing the emerging opportunities to support each other during the day. Occupational therapist Tina said: *‟Yes, and sometimes it is because as specific [situations] occur, we support each other […] It feels like it is an open climate. But there are no structured opportunities for … But we do take those opportunities when we see each other daily.”*

Overall, this highlights the importance of regularly handling staffs’ needs for reflecting, emplotting and sharing their rationale for actions to get peer support, whether by establishing fora for this as part of approved practice, or by the rare occurrence of leaving reasonable margins for informal NR.

Informally engaging in NR contributed to handling tacit and unpredictable demands of an ever-changing practice. When the procedures of the approved practice turned out to be insufficient, the NR were a resource staff used as an invisible, disregarded practice. Since the NR made staff conversant with the other professions’ everyday ways of working, unpredictable events could be communicated based on that understanding, hence better attuned to the routines. Furthermore, the analysis showed how participants spent much time establishing connections between people and information in and outside the ward to ensure continuity, through jointly constructing a reasonable narrative to guide decisions and action. As the OT Alicia said: *“Everything we do is not visible on the ward. What does it look like at home? How’s the family situation? Technical aids deliveries. What’s the situation with the cat?/ … /but that we have to communicate and make plans about.”*

Although identifying both some settled routines and some less successful efforts to establish new fora to create opportunities for fostering NR in the approved practice, these arrangements were seldom enough for keeping up with the demands of everyday healthcare work. Furthermore, the tendency to let medical issues dominate these occasions risked undermining the NR. As a result, these needs were met through forming a disregarded practice. Some staff enacted informal routines placed somewhere between approved and disregarded practice. Still, when the needs catered for in the disregarded practice are not generally and systematically acknowledged as a substantial part of healthcare work, eventually there may be consequences on the other arenas of practice, affecting the interactions and activities with the patients in the clinic frontstage.

### Narrative relations regarding four arenas of healthcare practice

The latter part of the analysis process led to insights about four arenas of practice where NR are adopted and enacted. We developed a graphical illustration built on the core theme continuum, and the theoretical resources to visualize these findings. Depicting the continuums as two intersecting axes provides a visual interpretation of four arenas of healthcare practice where NR are adopted and enacted (Figure 1). The figure shows how the NR are positioned in the arenas and their relation to the arena boundaries.

An arena is distinguished by a) the roles of the people involved, the types of relations between them, including the spatial context of these relations (i.e., *clinic frontstage* and *clinic backstage*), and b) whether the interactions are part of the *approved practice* of healthcare or performed as a *disregarded practice* – the conceptual distinction identified through the analysis, referring to how activities are approved and respected. It follows that an arena is not merely a physical place, nor a purely relational situation separated from spatiality. Moreover, the dividing lines in the figure should be understood as a simplifying—but not a simplistic—device (Mol & Law, 2002). Hence, the arenas are not discrete entities but rather exist on the continuums, admitting the communication between the arenas and the more complex reality of healthcare practice. Given that the figure’s axes represent continuums, the boundaries between the arenas must be understood as constructed and fluid. However, as these boundaries may inhibit the NR to reach between arenas, NR also have a transboundary function in interconnecting arenas of practice, thus defying these boundaries and supporting continuity and coherence.

## Discussion

Recent literature on person-centred care widely recognizes the importance of fostering a person-centred culture and of cultivating communication (McCormack et al., 2021; Santana et al., 2018). The focus on NR in our findings contributes with new insights about the role of narration in such efforts, both when referring to communication with patients and among staff. The notion of communication may be understood as merely transmitting medical information and facts, a risk that is enhanced in settings with a dominating biomedical focus. Negotiating different interpretations through NR allows for communication between biomedical interpretations and other perspectives. Still, the tension remains between establishing procedures for NR as part of the approved practice versus accepting that it may be impossible to settle some needs by formalities. In the findings, we may see a tendency towards a desire to translate narrative reasoning to fit organized forms or a logico-scientific structure, as described by Bruner in the theory of two modes of thought (Bruner, 1986). Since these forms of reasoning per definition are irreducible to one another, narrative reasoning may always find ways to escape such efforts. Instead, the findings may illustrate a consistent tension between the need for narrative meaning-making and the desire to organize and control such matters, and, furthermore, how NR help to make this tension visible. Here, we also want to point out a difference between the terms we have chosen and the constructs of formal and informal organization which has been employed in sociology for decades (Blau & Scott, 1962; Selznick, 1948). Approved practice may overlap with the formal organization but does not need to be equivalent to it. Formal definitions and policies are still subject to interpretation and practical application—a gap made visible by using the term approved practice. Following this line of thought, approved practice may also hold characteristics of informal organization, which is a reason for using other terms.

The findings of this study contribute to demonstrate the power of NR and the understanding of their function in relation to the multiple meanings and interpretive possibilities embedded in the personal, emotional, or existential matters dealt with in everyday healthcare practice. Such concerns of human life may continuously resist being treated as definite and unambiguous matters and NR may serve as a means for communicating such complexity and prevent over-simplification (Scholander et al., 2023). However, narrative is always selective; narrators pick out events and information they consider relevant to understand a situation and connect them through emplotment (Walker et al., 2020). The selection and connections assign significance to certain events while neglecting other, thus implying a certain interpretation and meaning. Thus, the narrative configuration represents one interpretation among others—a feature that requires awareness among people engaging in NR to prevent misunderstandings regarding what narratives represent. Such awareness seems crucial as a prerequisite for being able to constructively engage in NR and not risk mistaking narratives for realistic renderings of unambiguous facts. A possible understanding of our findings is that when narrative configurations are communicated through NR, negotiations between different interpretations are made possible, preventing definite and uniform interpretations. Inviting different perspectives to communicate enables insights into matters that are in fact multi-layered. This may be why the findings suggest that oral reports are considered most suitable for adopting NR; such interactions leave room for trying out and negotiating different interpretations compared to choosing a settled formulation in written records. The quote from Amanda about what is suitable to put in the records may potentially refer to the impossibility of negotiating interpretations and multiple perspectives in that form.

Based on the findings, we argue that instead of attempting to formalize NR, it is more important to raise awareness about the disregarded practice where NR often are adopted enacted and to acknowledge the value these practices add or the needs they meet in the local care setting. Essentially this is motivated from an ethical viewpoint, addressing matters related to how humane, person-centred, good quality healthcare comes about, enacted in everyday activities. Everyday narration as a means for human meaning-making is not a linear or, sometimes, even conscious undertaking. Hence, a disregarded practice may always exist to some extent and should be recognized in ongoing communication with other arenas of healthcare practice. Our findings shed light on the importance of this exchange between arenas where NR seem to play a crucial role for supporting person-centredness through their capacity to create and mediate everyday meanings regarding healthcare experiences, activities and decisions (Josephsson et al., 2022). Without means to pass boundaries between arenas, neither successfully elicited patient narratives nor other experiences or meanings mediated through narratives will be integrated fully in practice. Closely related to NR is the notion of *joint emplotment* (Fox & Brummans, 2019), referring to how staff construct and negotiate a joint understanding of patient situations through narration in backstage team meetings, thus located in the arena of approved practice in clinic backstage. What our findings add is insights about how NR extend beyond such bounded contexts as backstage meetings to also interconnect other arenas of practice.

We have deliberately focused on four main arenas of practice. Our ambition was to simultaneously sort out a complex reality by intentionally using simplifying devices (Mol & Law, 2002), yet acknowledging that this complexity cannot be fully resolved. Thus, the simplifying model has its shortcomings, but still illustrates how arenas are separated by constructed boundaries, how the NR may cross these boundaries to link arenas but also how NR may be obstructed by these boundaries. During the analysis process, we noticed other possible relational contexts such as interaction between professional groups, external organizations or even dyadic, individual relationships. All such bounded entities were not analysed and reported.

The findings imply that it may be valuable in any care setting to reflect on how existing arenas are negotiated and to explore to what extent these are interconnected. Here, the graphical illustration (Figure 1) may serve as a frame for reflection on how NR are adopted and enacted in local clinics, although further research is needed to develop and examine such application. Acknowledging NR in any arena may help to bridge boundaries. Although we identified several situations when engaging in NR was instinctively practiced for the purposes of upholding foundational qualities in healthcare practice, this competency seemed to be mostly tacit. This oblivious use of narration made the NR vulnerable to disruptions by the procedures of the approved practice focusing mostly on medical parameters. An implication of these findings is the importance of raising awareness about the occurrence of NR and the transboundary opportunities embedded in them. So instead of aiming for transforming one arena into another, the goal may rather be about moving from an oblivious and overlooked disregarded practice to acknowledging the disregarded level as a layer of healthcare practice—not for unreserved approval of all disregarded activities, but to better enable appraisal of this layer and the possible consequences on issues such as patient safety or person-centredness. This is also why a broader understanding of narrativity, such as NR, is a useful perspective in relation to person-centred care; narration deals with the ambiguity and uncertainty embedded in everyday practices—not by creating order in a paradigmatic sense (Bruner, 1986) but by creating order and coherence in a narrative sense, without eliminating the versatility embedded in the everyday experiences.

Our study is not without limitations, and we would like to address some of them. The findings mainly centre around the clinic backstage. Although our focus of this study was the staffs’ experiential knowledge, the divisions may have looked different if built on data from the patients’ perspectives. As others before us have emphasized, person-centredness is underpinned by values of respect for people and relations, which entails service users and others significant to them, as well as all care providers (McCormack et al., 2021). This study represents the care providers’ perspectives and does not claim to represent other perspectives. However, as shown in the findings, the arenas are not isolated from each other and what happens in one will affect how people can operate in the others. Better understanding of the clinic backstage helps understand the consequences in the frontstage; if NR function to bridge the arenas, contributing to uphold continuity and trustful relations, and preventing simplistic understandings of people and situations as previously described (Scholander et al., 2023) this certainly benefits the clinic frontstage and patients.

Our findings are based on data generated on one ward and do not claim to provide generalizable dictates applicable to all healthcare contexts. The balance between disregarded and approved practice may vary greatly between different sites, as well as which people or principles eventually decide what is granted the status of approved practice. There may be various reasons for this, including clinic area, local culture, assumed outcome measures or financial incentives.

Finally, this study has not investigated the underlying processes regarding what activities ultimately obtain the status of approved practice. Who or what regulates what is approved and prioritized, and how are divergent interests negotiated in practice? This merits further exploration in future studies.

## Conclusions

This study offers nuanced and situated insights into NR in staffs’ everyday work on a geriatric ward, grounded in their experiential knowledge of practice. The findings present a core theme showing how NR can be adopted as part of approved practice as well as enacted in a tacit disregarded practice to respond to the unpredictability and variability embedded in the everyday events on a geriatric ward. By distinguishing different arenas of practice, the findings show how NR are negotiated around the boundaries between arenas, either traversing them due to their transboundary potential and thus enhancing coherence and continuity between arenas, or being hindered by them, resulting in fragmentation from reinforced boundaries. Furthermore, the findings make visible tensions embedded in the continuums between the different arenas. These tensions may not be resolved by NR. However, through NR such tensions may become visible. Consequently, NR matter by offering a means to approach and respond to such tensions in an ongoing communication between positions pulling in different directions. Ultimately, this is about involving staff in the process of continuously evolving practice rather than expecting to reach a conclusive and unambiguous settlement of how healthcare work should be conducted. Instead of seeking to transform a fluid disregarded practice to the shape of a square approved practice, a more effective approach might be to acknowledge the disregarded NR as a vital and distinctive part of healthcare practice, and continuously cultivate an awareness of NR and what they express.

## Data Availability

The data that support the findings of this study are available on reasonable request from the corresponding author. The data are not publicly available due to privacy or ethical restrictions.
